# Investigating Associations Between Changes in Mobile Phone Use and Emotions Using the Experience Sampling Method: Pilot Study

**DOI:** 10.2196/formative.8499

**Published:** 2018-06-18

**Authors:** Suzanne Roggeveen, Jim van Os, Kelly Bemelmans, Mikal van Poll, Richel Lousberg

**Affiliations:** ^1^ Department of Psychiatry and Psychology Maastricht University Maastricht Netherlands; ^2^ Brain Center Rudolf Magnus Department of Psychiatry University Medical Center Utrecht Utrecht Netherlands; ^3^ Institute of Psychiatry Department of Psychosis Studies King's College London, King's Health Partners London United Kingdom

**Keywords:** mobile phone, experience sampling method, emotions, affect, concentration

## Abstract

**Background:**

The use of mobile phones has become, especially for young people, an integrated part of everyday life. Using the experience sampling method (ESM) may provide further insight on the association between mobile phone use and mental health.

**Objective:**

The objective of this study was to examine associations between mobile phone use and subtle changes in mental state.

**Methods:**

The ESM-based PsyMate app was installed on the mobile phones of 2 healthy 20-year-old participants. Over a period of 3 months, participants rated their mental states at 10 semirandom moments in the flow of daily life. Each assessment included present state emotions, environmental circumstances, and phone use.

**Results:**

Multilevel regression analyses indicated that an increase in mobile phone use was associated with a small increase in negative affect (particularly feeling bored and feeling lonely; *P*<.001) and small decreases in positive affect (*P*=.002) and concentration (*P*=.001). Treating the data as 2 separate N=1 studies revealed that the association with negative affect was present in both participants, whereas the associations with positive affect and concentration were evident in only 1 of the 2 participants.

**Conclusions:**

This pilot study suggests that mobile phone use may be associated with person-specific and group-level changes in emotional state. A larger study is required to study these associations, possible causality, and factors driving underlying heterogeneity in the pattern of associations.

**Trial registration:**

ClinicalTrials.gov NCT03221985; https://clinicaltrials.gov/ct2/show/NCT03221985 (archived by WebCite at http://www.webcitation.org/6zJnp61Wj)

## Introduction

Given widespread use of mobile phones, research has been conducted to investigate possible side effects. For example, mobile phone use is associated with cyberbullying [1] and slower response while driving [2]. Furthermore, mobile phone use may be excessive or even compulsive, which was coined as “problematic mobile phone use” [3]. At least 11 different questionnaires have been used to test the construct of mobile phone use addiction [3-14], mostly based on criteria for dependence as described in the *Diagnostic and Statistical Manual of Mental Disorders, Fourth Edition, Text Revision* (DSM-IV-TR) and now the DSM-5, which has comparable dependence criteria [15]. The psychological mechanism driving mobile phone addiction, dubbed nomophobia (the fear of being out of mobile phone contact), was proposed in 2014 [16]. The related fear of missing out (FoMO) was defined as “a pervasive apprehension that others might be having rewarding experiences from which one is absent” [17,18]. In addition to a possible addictive component, associations have been reported between mobile phone use and depression, anxiety, stress, and sleep problems [7,11,19-24]. Estimates of problematic mobile phone use across these different outcomes differ widely, ranging from 0% to 38% [25].

Problematic mobile phone use may be more prevalent in people who have certain personality traits [26]. Lower levels of self esteem were consistently associated with problematic mobile phone use across different studies [3,20,27,28]; higher levels of extraversion were associated with more frequent mobile phone use [3,29].

Although the above studies are examples of a growing body of work examining associations between mobile phone use and psychological constructs, the relation remains uncertain and research focusing on existing categories of psychopathology is inconclusive [30]. Therefore, in this pilot study, we addressed the more basic question of whether mobile phone use would impact daily life variation in core emotions, moving away from psychological constructs like personality traits or diagnostic categories. Given the highly dynamic process of mobile phone use, varying from moment to moment in daily life, the methodology used should be capable of capturing this dynamic process.

Studies in this area usually use traditional retrospective questionnaires that are associated with several well-known forms of bias and cannot capture dynamic variation of mental states associated with daily life phone use [31]. A data acquisition method which is relatively free of these kinds of bias is the experience sampling method (ESM) [32]. ESM is a valid and reliable method which can contribute to the understanding of the relationship between environmental phenomena and mental experience [33]. Another advantage of this method is the ability to detect possible subtle (unconscious) changes in affect in the context of daily life in relation to mobile phone use. There are also other mobile phone apps using ESM [34,35]. A recent Australian study examined the use of such an app for monitoring well-being in context and in real time, including personalized feedback [34].

In order to pilot the use of ESM in the context of mobile phone use, an ESM pilot study (N=2) was conducted in order to examine associations between mobile phone use and subtle changes in mental states. In ESM studies, participants are beeped at semirandom moments to record their in-the-moment emotional and behavioral states several times during the day (usually 6 to 10 times) [33]. In addition, the actual circumstances (where and with whom a participant is) are also rated. This dynamic repeated measure design allows for prospective modeling of behavioral and affective dynamic patterns during the day as well as for examining associations with context [33].

Guided by previous literature, the following hypotheses were postulated: (1) a general increase of phone use will be associated with higher scores on negative affect, lower levels of positive affect, and lower levels of concentration [21,36] and (2) whenever a person cannot engage in mobile phone use, increased levels of negative affect will ensue.

## Methods

### Participants

Recruitment took place in September 2016 using posters distributed at Maastricht University Medical Centre in Maastricht, The Netherlands. Inclusion criteria were aged 20 to 25 years, healthy, good understanding of the Dutch language, and mobile phone use. Exclusion criteria were a psychiatric diagnosis and pregnancy. Two healthy, female volunteers, both aged 20 years and students at Maastricht University, were enrolled. Participants were frequent mobile phone users who were used to carrying a mobile phone with them at all times and did not experience their phone use as problematic. Participants were compensated €150 (US $177).

### Procedures

#### Briefing

During the briefing, participants were helped to download the ESM PsyMate app on their mobile phone (from the App Store or Google Play). They were instructed on how to use the app. It was stressed that participants should not change their daily life routines; they were asked to carry their mobile phone with them at all times in order to miss as few beeps as possible. In addition to the ESM scheme, participants completed separate morning and evening questionnaires. On the morning questionnaire, before the first beep of the day, participants were asked to answer some questions about the quality of sleep and the location of the mobile phone in the past night (Multimedia Appendix 1). In the evening, after the last beep, some additional questions were asked about the use of the PsyMate app and its possible impact on the participant (Multimedia Appendix 1). Morning and evening questionnaires were presented to the participants every day of the study. At all times during the study, a researcher (SR) was available for questions. After the briefing session, the study period of 3 months started.

#### Data Collection

In order to collect ESM data, the PsyMate app [37] was installed on the personal mobile phone of participants. Data collection took place over a period of 3 months. The PsyMate app emitted 10 beeps per day at semirandom intervals in each of ten 90-minute time blocks between 7:30 am and 10:00 pm. The app worked independently of internet connectivity. After each beep, participants were asked to answer the ESM items as soon as possible (thus capturing information about the in-the-moment state). Items should be answered within 15 minutes in order to safeguard real-time assessment [38]. There were 4 positive affect ESM items (cheerful, mentally fit, relaxed, and globally feeling well), and 6 items indexed negative affect (irritated, bored, lonely, gloomy, stressed, and worried). Next, current context and activities (physical activity, daily life activities, persons present) as well as physical items (tired, level of concentration, and pain) were rated. Finally, mobile phone use since the last beep (frequency, frustration when not able to use the mobile phone) was measured. An overview of ESM items is presented in Multimedia Appendix 1. The affect items were rated on a 7-point Likert scale, ranging from not at all to very much. Mobile phone use was rated as follows: at each beep, the participant was asked to give an estimate of the frequency of mobile phone use between the last beep and the present beep. Zero represents no use, 1 represents once, 2 represents 2 to 5 times, 3 represents 5 to 10 times, and 4 represents more than 10 times. Over the total period, participant 1 had a mean between-beep mobile phone use of 1.72 (SD 1.06) and participant 2 a mean of 0.81 (SD 0.98).

After the 3-month ESM period, a debriefing session was held with both participants. Participants were asked to what degree the past 3 months were representative of their daily life and to what extent PsyMate had interfered with their normal routines.

#### Data Reduction

App data were directly transferred to an internet cloud when internet connectivity was available. When there was no connection to the internet, data were temporarily stored on the mobile phone. The data of both participants were directly extracted from the cloud and imported in an SPSS Statistics (IBM Corp) datafile.

PsyMate studies have shown that positive and negative affect (PA and NA, respectively) items can be reliably (Cronbach alpha>0.7) and consistently [33] scaled into 2 factors: a PA and an NA factor.

### Analyses

Data on momentary mental state at beep moment *t* and level of phone use between the last beep (*t–1*) and the present (*t*) were retrieved in order to model affect parameters as the dependent and phone use as the independent variable in the analyses.

Statistical analyses were conducted with SPSS 24.0 (IBM Corp) [39]. The data were hierarchically structured, since multiple assessments were performed and clustered within days that in turn were clustered within participants. Multilevel random regression analyses were used to test the relationship between mobile phone use and affect, taking the hierarchical structure into account. In order to model time effects, beep number and beep time interval were incorporated as covariates in all analyses. In order to correct for interdependence of the data over successive beeps, an autoregressive (AR1) covariance structure was selected.Since there were 2 participants, allowing for investigation of person-specific patterns, data were also analyzed as 2 N=1 studies. To this end, linear regression analyses, using affect as dependent parameter and mobile phone use as predictor, were performed separately for participant 1 and 2. In these regression analyses, beep number and beep time interval were also incorporated as covariates. A 2-sided *P*<.05 was considered statistically significant.

### Ethics Statement

Approval was obtained from the medical ethics committee of the Academic Hospital Maastricht on May 19, 2016. All participants provided written informed consent.

## Results

Participant 1 used the PsyMate app during 96 days and responded to 399/960 beeps (41.6%). Thus, on average 4.2 out of 10 beeps each day were completed. Participant 2 completed 506/930 beeps (54.4%) or 5.4 beeps out of 10 each day. Guided by previous work with this ESM scheme, a response of 30% to the ESM beeps was considered the minimum for inclusion in the analysis [38].

In order to model change in affect from beep to beep in relation to mobile phone use, an *a priori* limit was set on days with at least 4 completed beeps. In participant 1, 62 of 96 days met this requirement (65% of all the days), with an average of 5 completed beeps per day. In participant 2, 78 out of 93 days (84% of all days) met the requirement, with an average of 6 beeps per day. Setting the *a priori* limit caused an ESM data reduction of 19% in participant 1 and 8% in participant 2.

Table 1 shows that responding to PsyMate beeps generally was not rated as impacting mood or normal phone use. One change was that normally participants would not carry their phones with them at all times, but because of the study they now did so. Furthermore, participants noted that it was tempting to take a look at their phone after a PsyMate beep some of the time. A difference in response between the 2 participants can be noted in FoMO: participant 1 experienced this feeling more often than participant 2.

In Table 2, an overview of the results of multilevel (results of the 2 participants together) and linear (results per participant) regression analyses is provided. The frequency of mobile phone use between the previous and the current beep was modeled as predictor of current affect variables. In the combined dataset, higher levels of mobile phone use were associated with more NA and less PA. Decomposing NA and PA into their separate constituents revealed that bored and lonely stood out in strength of association among the NA items, whereas cheerful, relaxed, and globally feeling well stood out among the PA items. Item concentration was also negatively associated with higher levels of phone use. Analyzing participant data separately revealed that the association between frequency of phone use and NA was consistent across participants. The association with PA was consistent across the 2 participants, although only significant in participant 1. Cohen *d* was 0.14 for negative affect and 0.11 for positive affect.

The second hypothesis, that when a participant was not allowed to or capable of using a mobile phone, she would report more negative affect parameters, could not be tested due to a lack of data. For example, participant 1 only reported no phone use in 56 beeps, of which only 8 could be attributed to being permitted to use a mobile phone.

**Table 1:** Results from evening questionnaires of participant 1 (n=52) and participant 2 (n=84).

Question	Participant 1, mean (SD)	Participant 2, mean (SD)
Responding to PsyMate beeps has influenced my mood.	1.54 (1.24)	1.82 (0.88)
Without PsyMate, I would have done other things today.	1.12 (0.83)	1.02 (0.153)
After responding to PsyMate beeps, I used my mobile phone.	2.98 (1.85)	4.11 (1.73)
PsyMate has influenced my normal phone use today.	2.17 (1.32)	1.44 (0.91)
Today, I experienced fear of missing out.	3.62 (1.57)	1.39 (0.70)

**Table 2:** Estimates of mobile phone use and affect.

Characteristic		Participant 1 and 2		Participant 1		Participant 2		
		Beta coefficient of mobile phone use	*t* value	Beta coefficient of mobile phone use	*t* value		Beta coefficient of mobile phone use	*t* value
**Negative affect**		0.47	4.07	0.67	2.54		0.33	2.62
	Irritated	0.07	1.71	0.06	0.72		0.08	1.94
	Bored	0.22	5.37	0.31	3.87		0.14	3.30
	Lonely	0.12	4.60	0.22	3.89		0.05	1.76
	Sadness	0.05	1.44	0.05	0.62		0.03	0.66
	Stressed	0.04	1.19	0.01	0.09		0.01	0.15
	Worried	0.03	0.88	0.03	0.43		0.04	0.76
**Positive affect**		–0.29	–3.09	–0.41	–1.90		–0.08	–0.69
	Mentally fit	0.00	0.14	0.04	0.67		0.02	0.74
	Cheerful	–0.10	–2.72	–0.16	–2.16		–0.04	–0.85
	Relaxed	–0.09	–2.39	–0.19	–2.58		–0.01	–0.27
	Globally well	–0.11	–3.37	–0.09	–1.38		–0.06	–1.67
**Other**								
	Tired	0.05	1.18	–0.06	–0.69		0.06	0.89
	Concentrating	–0.13	–3.26	–0.26	–3.54		–0.05	–0.97

## Discussion

### Principal Findings

The main findings of this study: an increase in mobile phone use is associated with a small increase in NA (particularly feeling bored and feeling lonely) and small decreases in PA and concentration.

In this N=2 pilot study, participants answered questions in the PsyMate app about their mental state and mobile phone use over a 3-month period. The goal was to investigate whether an association between the two could be demonstrated as a prelude to a larger study. Guided by previous work, it was hypothesized that a general increase of phone use would be associated with higher scores on NA, lower levels of PA, and lower levels of concentration [21,36]. The results show that these hypotheses were confirmed. Although all of the NA items showed a positive association, the NA items lonely and bored stood out. Based on the literature, it was also expected that other components of NA such as sadness would be significantly associated with mobile phone use. This item was directionally associated with mobile phone use but below the conventional limit of significance. The significant decrease of PA after an increase of phone use was seen in 3 out of 4 components of PA. While the association with NA was replicated across the 2 participants, the association with PA and concentration appeared to be person-specific. Person-specific moderators may play a role, such as level of self-esteem and extraversion that have been linked to problematic phone use, as well as other personality traits [40]. In the current analysis, participant 1 reported more FoMO. The experience of FoMO may also underlie person-specific results in the association between mental state and mobile phone use.

It was not possible to investigate the hypothesis that not being permitted to use a mobile phone would result in higher levels of NA due to a lack of data resulting in lack of variability.

This study indicates that in-the-moment associations between mobile phone use and affect may be subtle and difficult to report using retrospective reports from cross-sectional questionnaires. Participants in this study appeared to differ in their global and in-the-moment reports. If the outcomes of this study can be replicated in a larger study population, combining group-level and person-specific approaches of analysis, it may be easier to develop person-specific and evidence-based approaches for problematic mobile phone use.

### Limitations

This was a pilot study and results must be considered preliminary. A larger study is required to further study the associations described in this analysis. Second, based on the results from Table 1, it may be hypothesized that the use of a mobile phone ESM app to collect the data may have influenced mobile phone use to some extent, with a potential for bias. To what degree this may impact the results has to be investigated in future studies. Third, in this pilot the level of mobile phone use (in minutes) was estimated at each beep by the participants themselves. It would have been more accurate to also measure phone use digitally in duration and type (distinguishing between email and social media, for example).

### Conclusion

In future research, the above considerations need to be taken into account. In addition, there are some other recommendations. First, it would be instructive to investigate the effects of mobile phone use on the quality of sleep, as an association was suggested in previous studies [21,36]. In this study, the questionnaire was included but analyses could not be performed due to lack of variability in the data. Second, as observed in the evening questionnaire of this ESM pilot, FoMO can vary greatly between individuals and it would be desirable to add this question to the beeps.

## Appendix

Multimedia Appendix 1 PsyMate questionnaires.

## Abbreviations

ESM: experience sampling method
FoMO: fear of missing out
NA: negative affect
PA: positive affect
