# Supplementary material for: Investigating Associations Between Changes in Mobile Phone Use and Emotions Using the Experience Sampling Method: Pilot Study
Source: JMIR Form Res. 2018 Jun 18;2(1):e12. doi: 10.2196/formative.8499 (PMC6334687; doi:10.2196/formative.8499)
Supplement: Multimedia Appendix 1 [file formative_v2i1e12_app1.pdf]

Multimedia Appendix 1. PsyMate questionnaires.

**Domain**	**Description**	**Scale**
affect	I feel cheerful	1=not 4=moderate 7=very
	I feel irritated	1=not 4=moderate 7=very
	I feel mentally fit	1=not 4=moderate 7=very
	I feel bored	1=not 4=moderate 7=very
	I feel relaxed	1=not 4=moderate 7=very
	I feel lonely	1=not 4=moderate 7=very
	I feel sad	1=not 4=moderate 7=very
	I feel stressed	1=not 4=moderate 7=very
	I am worried	1=not 4=moderate 7=very
	Overall I feel well	1=not 4=moderate 7=very
context	Activity, I am …	1=lying 2=sitting 3=walking 4=biking 5=sports 6=other
	At the moment I am …	1=eating/drinking 2=hygiene 3=work/school 4=resting 5=PC/tablet 6=phone 7=household 8=TV 9=face to face conversation 10=going out 11=other
	I am doing this because …	1= general health 2=fun 3=distraction 4=obligatory 5=other
	This costs effort	1=not 4=moderate 7=very
	I would rather do something else	1=not 4=moderate 7=very
	At the moment I am at…	1=home 2=someone else's home 3=at work/school 4=public space 5=on the road
	At the moment I am in the company of…	1=nobody 2=partner 3=family 4=friends 5=colleagues 6= acquaintances 7=strangers
	This is agreeable	1=not 4=moderate 7=very
other	I am tired	1=not 4=moderate 7=very

	I am concentrated	1=not 4=moderate 7=very
	I have physical complaints	1=not 4=moderate 7=very
	Location of pain	1=head 2=neck 3=back 4=abdomen 5=hands 6=other
	These complaints have … since the last beep	1=reduced 2=stayed the same 3=increased
dependency	I have used my phone since the last beep	0=not 1=once 2=2-5x 3=5-10x 4=>10, when 0
	I have not used my phone because…	1=forbidden 2=not possible 3=no need
	It was frustrating to not be able to use my phone	1=not 4=moderate 7=very
	The last time I used my phone was …	1=<5min ago 2=5-15min ago 3=15-45min ago 4=>45 min ago
evaluation	This beep disturbed me	1=not 4=moderate 7=very

Morning questionnaire

	Item	Scale
1	How long did it take to fall asleep yesterday evening?	1=0-5min 2=5-15min 3=15-30min 4=30-60min 5=1-2hours 6=2-4hours 7=>4hours
2	How often did you wake up during the night?	0->5
3	I slept well	1=not 4=average 7=very
4	How many hours did you sleep?	1=<3hours 2=4-5hours 3=6hours 4=7hours 5=8hours 6=9hours 7=>10hours
5	How many alcoholic bevarages did you drink yesterday evening?	1=0 2=1-2 3=3-5 4=6-10 5=>10

Evening questionnaire

	Item	Scale
1	Answering the PyMate™ beeps has influenced my mood	1=not 4=average 7=very
2	Without PsyMate™ I would have done other things today	1=not 4=average 7=very
3	After answering the Pysmate beeps I have used my mobile phone	1=not 4=average 7=very
4	The PsyMate™ has influenced my normal phone usage today	1=not 4=average 7=very
5	Today I experienced fear of missing out	1=not 4=average 7=very
6	Where was my phone mostly today	1=on/against my body 2=bag 3=elsewhere whithin easy reach 4=out of reach 5=not with me
